# Book Reviews

**Published:** 1901-03

**Authors:** 


THERAPEUTICS: ITS PRINCIPLES AND PRACTICE. By HORATIO C. WOOD, M.D., L.L. D., Professor of Materia Medica and Therapeutics, and Clinical Professor of Diseases of the Nervous System in the University of Pennsylvania; and Horatio C. Wood, Jr., M. D., Demonstrator of Pharmacodynamics in the University of Pennsylvania. Octavo, pp. xxxiii. - 850. Eleventh edition, remodeled and in greater part rewritten. Philadelphia and London: J. B. Lippincott Co. 1900.

During the past twenty-five years Wood's treatise on therapeutics has enjoyed the confidence of the medical profession, both at home and abroad. When the first edition appeared it was believed to be the first book in any language, and known to be the first in the English language, to make the physiological study of drugs the essential of practical therapeutic teaching. Nearly every work that has appeared since 1875 has been written upon the same basis.

In the ten editions that have already appeared, whatever of progress which has occurred between each has been carefully noted, so that taking the work as a whole from its first edition to its last it has not only kept abreast of the periods of its issuance, but in a certain sense and in some respects it has been in advance of accepted teachings.

Some changes have been made in the present edition consisting chiefly in condensing certain portions of the work, in eliminating others, in a careful selection of language with a view to obtain conciseness but not to sacrifice clearness, and in adding articles on a number of new drugs. The author's son, Dr. Horatio C. Wood, Jr., has been associated with him in the revision of this treatise, which shows everywhere careful work by master hands. We have heretofore had occasion to speak in terms of commendation of this work and we now reaffirm our previously expressed opinions with compound interest.

---

A TEXT-BOOK OF THE DISEASES OF WOMEN. By HENRY J. GARRIGUES, A. M., M. D., Gynecologist to St. Mark's Hospital, New York City. Octavo volume of 756 pages, with 367 illustrations. Third edition, thoroughly revised. Philadelphia and London: W. B. Saunders & Co. 1900. [Price, cloth, $4.50 net; sheep or half Morocco, $5.50 net.]

The author of this treatise has been long known to the profession of medicine as one of the most painstaking gynecological teachers. His text-book, therefore, was well received when it first appeared, not only by the medical press, but by teachers and practitioners of medicine throughout the country; hence, it is not surprising that the earlier editions promptly became exhausted.

The demand for the third edition has enabled the author to revise the entire work in a thorough-going manner, bringing its teaching down to the immediate present. All this has been done in the most conscientious way, so that the present volume represents the gynecologic practice of today quite as completely as any of its contemporaries in this field of medicine. Whatever has ceased to be of importance has been eliminated and much new material in text and illustrations has been added. One of the chief advantages of this text-book is its eminently practical character. It, therefore, appeals to student and practitioner alike as a safe guide in the amphitheater or library. Moreover, the simplicity and directness of its language make it one of the most valuable text-books for beginners that has yet appeared. It is not overloaded with theoretical discussions, but it is strong in pathology, diagnosis and treatment. We most heartily commend it to student and general practitioner.

---

PROGRESSIVE MEDICINE. Volume IV. 1900. A Quarterly Digest of Advances, Discoveries and Improvements in the Medical and Surgical Sciences. Edited by HOBART AMORY HARE, M. D., Professor of Therapeutics and Materia Medica in the Jefferson Medical College of Philadelphia. Octavo, handsomely bound in cloth, 428 pages, 69 illustrations. Philadelphia and New York: Lea Brothers & Co. [Per annum, in four cloth-bound volumes, $10.co.]

The contents of this volume includes articles as follows: Diseases of the Digestive Tract and Allied Organs, the Liver, Pancreas and Peritoneum, by Max Einhorn; Genito-urinary Diseases and Syphilis, by William T. Belfield; Fractures, Dislocations, Amputations, Surgery of the Extremities, Orthopedics, by Joseph C. Bloodgood; Diseases of the kidneys, by John Rose Bradford; Physiology, by Albert P. Brubaker; Hygiene, by Henry B. Baker, and Practical thera-peutic referendum, by E. Q. Thornton.

In Einhorn's article the latest methods of medicating the stomach will be found recorded, as well as many valuable hints on medical diagnosis and treatment. Bloodgood's article is most instructive, and among other things, he devotes considerable space to gonorrheal arthritis. The therapeutic referendum is much enlarged. Dr. Baker's article is a practical one, dealing with the causation of diseases due to toxins, their modes of communication and methods of control.

This is one of the most useful volumes of this very excellent series.

The book is well illustrated, its letter press is clear and distinct, while its general make-up is attractive.

---

A TEXT-BOOK OF HISTOLOGY, INCLUDING MICROSCOPIC TECHNIC. By A. A. BÖHM, M. D., and M. VON DAVIDOFF, M. D., of the Anatomical Institute in Munich. Edited, with extensive additions to both text and illustrations, by G. Carl Huber, M. D, Junior Professor of Anatomy and Director of the Histological Laboratory, University of Michigan. Authorised translation from the second revised German edition, by Herbert H. Cushing, M. D., Demonstrator of Histology and Embryology, Jefferson Medical College, Philadelphia. Octavo, pp. 501, 351 illustrations. Philadelphia and London: W. B. Saunders & Co. 1900. [Price, $3.50 net.]

This is the first American from the second German edition and presents in an approved fashion, the fascinating study of intimate structure of the tissues of the body. Beginning with an introductory chapter on microscopic technic, the author passes to the consideration of general histology in which he first treats of the cell and then of the tissues. In this way the first 164 pages of the book are utilised. In the second section of the treatise special histology is taken up and is considered under the following separate heads: I. Blood and blood-forming organs, heart, bloodvessels, and lymphvessels; II. Circulatory system; III. Digestive organs; IV. Organs of respiration; V. Genito-urinary organs; VI. The skin and its appendages; VII. The central nervous system; VIII. Eye; IX. Organ of hearing; X. Organ of smell, and XI. General considerations of the special sense organs.

From this it will be observed that the scheme and scope of the work is based upon modern teaching and research. It may be added, also, that it is exhaustive without being tedious, that it is concise without doing violence to essentials, and, in short, is one of the best text-books on histology for students and investigators, as well as teachers, that recently has appeared. The illustrations are especially worthy of note and the translator has preserved, to a remarkable degree, the original ideas of the author and at the same time given us smooth-reading English. The American editor has added notes here and there, where necessary, to make the work conform to the present teaching in American laboratories. It is a text-book deserving of warmest commendation.

---

Physical Diagnosis of Diseases of the Chest. By Richard C. Cabot, M. D., Physician to Out-patients, Massachusetts General Hospital; Assistant in Clinical Medicine, Harvard Medical College. Octavo, pp. xv.—310. 142 illustrations. New York: William Wood & Co. 1900. [Price, $2.50.]

The author of this excellent book modestly announces that it is intended for students and, so far as he is aware, contains nothing original. He further states that he has written it because he has not been able to find any small work upon the subjects that does not contain glaring errors, the correct books being too large and the small ones are out of date. This is an argumentum ad judicium, and is ample reason for the appearance of the work. There is room for it; it is well written; the author knows his subject; he imparts his knowledge clearly,—and this is more than can be said of every medical book published nowadays.

There is no more important study, after the fundamentals, than that of diagnosis. The subtleties of disease that lurk within the cavity of the chest give forth a correct interpretation only to the trained eye, ear and touch. This work by Cabot, unless we are very greatly in error, will do much toward clearing up doubtful sounds from heart or lungs that have either been little understood or greatly misunderstood.

Austin Flint was one of the first American physicians to point out the importance of properly differentiating râles, friction sounds and murmurs, and to properly teach his pupils how to estimate their true value and significance. Cabot has taken up the work where Flint left it and has refined it beyond any teacher of the present day. His chapter on valvular lesions is altogether the finest bit of teaching on the subject we have ever seen, whether in text or illustration, and ought to be carefully studied by every physician. There are other chapters, especially the one upon auscultation, which might similarly be commended. Indeed, the book, as a whole, merits approbation and especially do its illustrations deserve praise. No intelligent student of diseases of the chest, whether undergraduate or practitioner of medicine, can afford to deny himself the possession of this book.

---

Appendicitis and its Surgical Treatment. With a Report of 185 Operated Cases. By Herman Mynter, M. D., Professor of Clinical Surgery in University of Buffalo, Buffalo, N. Y. Duodecimo, pp. 231. Third revised edition. Philadelphia: J. B. Lippincott Co. 1900. [Price, $2.00.]

When the first edition of this treatise was received the Journal reviewed it in extenso, expressing an opinion upon the several points of importance made by the author in his consideration of the subject. The second edition was partly destroyed by fire when the Lippincott house was burned in 1899, hence the Journal did not receive a copy.

In the third edition the general plan of the book has not been changed, except to omit the histories, which has very much reduced it in size. Instead of the histories of cases, which comprised such a large portion of the first edition, in this they have been classified according to their different clinical forms. The work is a valuable contribution to the literature of appendicitis and presents the strongest arguments on the surgical side of the question. There is constantly less reason for regarding this as a medical disease and, with accumulated experience, there is constantly more reason for regarding it as a surgical malady. Every surgeon will appreciate this book because of the strong personality of the writer and the stand he takes with reference to early operation.

---

A Manual of Materia Medica and Pharmacology. Comprising all Organic and Inorganic Drugs, which are and have been official in the United States Pharmacopœia, together with important allied species and useful synthetics. For students of medicine, druggists, pharmacists and physicians. By David M R. Culbreth, M. D., Professor of Botany, Materia Medica and Pharmacognosy in the Maryland College of Pharmacy, Baltimore. New (2d) edition. In one 8vo volume of 881 pages, with 464 illustrations. Philadelphia and New York: Lea Brothers & Co., Publishers. 1900. [Cloth, $4 50 net ]

This treatise attracted marked attention when its first edition appeared in 1896. It was adopted as a text-book by many of the colleges and has steadily kept favor with the profession. Among its advantages may be enumerated its convenient size, the vast information it contains, the clearness with which it is presented and the original methods adopted with reference to the order of the arrangement.

In this edition the general scope of the work remains practically the same; the author's aim to keep the basal or parental source paramount and to associate in regular order those substances which have a common or allied origin, has been adhered to. Again, the latest approved remedies are recorded, a more detailed statement of the physiological action of leading drugs is given, a more comprehensive account of poisons is presented and several other changes have been made, including a number of new illustrations, which serve to keep the work in the front rank of progress. It is a treatise that both students and teachers cannot fail to appreciate, because it easily takes rank among the best of its kind and is well adapted for use in the laboratory, class room and library.

---

A POCKET TEXT-BOOK OF DISEASES OF THE EYE, EAR, NOSE AND THROAT, for Students and Practitioners. By WILLIAM L. BALLENGER, M. D., Assistant Professor of Otology, Rhinology and Laryngology in the College of Physicians and Surgeons, Chicago, etc., and A. G. Wippern, M. D., Professor of Ophthalmology and Otology in the Chicago Eye, Ear, Nose and Throat College. Duodecimo, 525 pages, with 150 engravings and six full-page colored plates Philadelphia and New York: Lea Brothers & Co. 1900. [Price, cloth, $2.00 net; flexible red leather, $2.50 net.]

This is a practical work, treating of diseases of the eye, ear, nose and throat. It is abundantly illustrated, possessing advantages for students and general practitioners, as diseases of these organs are among the most common conditions which medical men are called upon to handle.

The book reflects the most advanced state of its subjects, both in theory and practice, in diagnosis and treatment. The nomenclature of diseases of the eye is much less complex than in the ordinary text-book, while those diseases of the organ which the general practitioner is expected to deal with, are fully described.

In the section on the ear the physiologic tests of hearing are given considerable prominence. Suppurative diseases of the middle-ear and mastoid are fully described. The mastoid operation is dwelt upon with marked detail, each step being given which will interest the experienced aurist.

Obstructed nasal respiration is fully described in its etiological relation to many nasal and naso-pharyngeal diseases. The symptoms of suppurative diseases of the accessory sinuses of the nose are explained from their anatomical relations to each other, and to the turbinated bones. The section on diseases of the throat is concise but not exhaustive.—W. S. R.

---

MODERN SURGERY. General and Operative. By JOHN CHAMBERS DACOSTA, M. D., Professor of the Principles of Surgery and of Clinical Surgery, Jefferson Medical College, Philadelphia; Surgeon to the Philadelphia Hospital and to St. Joseph's Hospital, Philadelphia. Octavo, pp. 1117. With 493 illustrations. Third edition, revised and enlarged. Philadelphia and London: W. B. Saunders & Co. 1900. [Price, cloth, $5.50 net; half morocco, $6.00.]

The general plan of this work, as established in its first edition, is still adhered to. This, in brief, is a presentation of the fundamental principles of surgery, together with a description of its chief operations and the most accepted methods of surgical technique. To facilitate this plan special surgery, such as pertains to ophthalmology, gynecology, etc., has not been considered. These have been left to the several special treatises, which is a most wise arrangement.

Taking advantage of the opportunity the author has made important additions to the work, such, for instance, as have grown out of improvements in surgical science, better methods of teaching, or the just criticisms of reviewers. Separately considered, each of these would have required but comparatively few changes, yet in the aggregate they have rendered necessary the addition of much new material. To meet this demand some obsolete matter has been eliminated, leaving the general size of the volume quite the same. Its simplicity and directness appeal to student and practitioner of medicine, who may herein find a safe guide to the proper diagnosis and management of surgical accidents and injuries.

The American Year-Book of Medicine and Surgery for 1901. A Yearly Digest of Scientific Progress and Authoritative Opinion in all Branches of Medicine and Surgery, drawn from journals, monographs and text-books of the leading American and foreign authors and investigators. Arranged with critical editorial comments, by eminent American specialists. In two volumes—Volume I, including *General Medicine*, Octavo, 681 pages, illustrated; Volume II, *General Surgery*, Octavo, 610 pages, illustrated. Philadelphia and London: W. B. Saunders & Co. 1901. [Per volume: Cloth, $3.00 net; half morocco, $3.75 net.]

This excellent year-book is again issued in two volumes as was done last year—one pertaining to medicine and the other to surgery. This arrangement has the advantage of convenience on the one hand and permits those who want the medical or surgical volume alone to obtain either without the increased expense of buying the other.

The arrangement of the volumes is excellent, the material is good, and the letter press superb. In the surgical volume we think it would be well to introduce illustrations more copiously, as these go a long way toward popularising such a work. We regard this as one of the best of the annual digests of medical literature.

---

A TEXT-BOOK OF THE PRACTICE OF MEDICINE. By JAMES M. ANDERS, M.D., Ph. D., LL. D., Professor of the Practice of Medicine and of Clinical Medicine in the Medico-Chirurgical College, Philadelphia; Attending Physician to the Medico-Chirurgical and Samaritan Hospitals, Philadelphia. Octavo, pp. 1263. Illustrated. Fourth edition, thoroughly revised. Philadelphia and London: W. B. Saunders & Co. 1900. [Price, cloth, $5.00 net; sheep and half morocco, $6.50.]

It is but a year since we presented a notice of the third edition of this text-book. The several editions have been exhausted with such remarkable rapidity that it leaves but little for the author to do between the disappearance of one and the demand for the next, except to modify the text here and there and to make the few additions that are necessary to keep it abreast of the date of issue.

Diseases of the digestive system have received additions and a number of subjects have been rewritten. The book remains substantially the same in size and is still one of the best treatises on the practice of medicine of recent issue.

A MANUAL OF HYGIENE AND SANITATION. By SENECA EGBERT, A. M., M. D., Professor of Hygiene in the Medico-Chirurgical College of Philadelphia. New (2d) and revised edition. In one 12mo volume of 427 pages, with 77 engravings. Philadelphia and New York: Lea Brothers & Co. 1900. [Price, cloth, $2.25 net.]

The appearance of a second edition of Egbert's work on Hygiene and sanitation proves the value of the book and the widespread

Nineteenth Annual Report of the State Board of Health of New York. Transmitted to the Legislature February 13, 1899. With accompanying maps. New York and Albany. 1900.

After an existence of twenty-one years the State Board of Health has been abolished in accordance with the recommendation of the governor, and a single commissioner appointed instead thereof, at an annual salary of $3,500. The State of New York, which should be first in everything that relates to the welfare of its citizens, has by this inexplicable action of its executive and legislative branches of the government turned back the wheels of progress for an unlimited period of time.

All interested in state medicine, public sanitation and general hygiene will be curious to learn what class of man who, is to act as expert in all these matters, can be obtained for the meager salary offered. From this action of the state government we presume that this will be the last report by the State Board of Health. These reports are two years behind—a tardiness that ought to have been remedied long ago.

---

Report of the Commissioner of Education for the Year 1898-99. Volumes I and II. Washington: Government Printing Office. 1900.

Volume I. is devoted principally to a consideration of the educational status in foreign countries. One chapter—XXII.—gives a list of confederate text-books in vogue from 1861 to 1865, which is amusing, to say the least. In it are given such titles as The Dixie Speller, The New Texas Spelling Book, The First Dixie Reader, The Southern Confederacy Arithmetic, The Louisiana English Grammar. The latter is "published by order of his excellency, Henry W. Allen, governor of Louisiana." One curious title is the Palmetto Dictionary. Among the extracts from some of these books we note the following: "This (United States) was once the most prosperous country in the world . . . The people are ingenious and enterprising and are noted for their tact in driving a bargain." Relating to the Southern Confederacy we note the following extract: "This is a great country. The Yankees thought to starve us out when they sent their ships to guard our seaport towns. . . . . The Southern people are noted for being high-minded and courteous."

---

TRANSACTIONS OF THE TEXAS STATE MEDICAL ASSOCIATION. Thirty-second Annual Session held at Waco, Texas, April 24-27, 1900. H. A. WEST, M.D., Secretary.

The book of annual transactions of the Texas State Medical Association always presents an excellent appearance in form and dress. This results from keeping an experienced secretary at the helm from year to year. An excellent feature of this number is a verbatim report of the proceedings, which takes up about 120 pages of the volume.

The association honored itself by electing Dr. B. E. Hadra to be president for the current year. Dr. Hadra is an experienced surgeon and an ornament to the profession of medicine. After the proceedings, a number of papers read at the meeting are published, which cover the several branches of medicine and surgery and furnish literature of interest.

---

A CLINICAL TREATISE ON FRACTURES. By WILLIAM BARTON HOPKINS, M. D., Surgeon to the Pennsylvania Hospital and to the Orthopedic Hospital and Infirmary for Nervous Diseases. Octavo, pp. 268. Illustrated. Philadelphia: J. B. Lippincott Co. 1900.

The literature relating to the treatment of fractures has been enriched of late, by the publication of several new works devoted solely to that branch of surgery. This exclusively clinical treatise will take front rank in the class of literature referred to. It everywhere bears the imprint of careful study.

The author, himself, is a master of his subject and he has had great clinical experience at the Pennsylvania Hospital. He describes the injury and its management in each particular case in a concise manner, scarcely using a superfluous word. It is a work for the man of some experience rather than for the student. It is copiously illustrated with admirable skiagraphs and numerous other engravings, selected from the vast collection at the Pennsylvania Hospital and from the Mütter Museum of the College of Physicians.

It is printed on heavy plate paper, and is as handsome a specimen of book-making as has been seen in many a day.

